# Supplementary material for: WordCommentsAnalyzer: A windows software tool for qualitative research
Source: F1000Res. 2018 Sep 4;7:536. Originally published 2018 May 3. [Version 2] doi: 10.12688/f1000research.14819.2 (PMC6137411; doi:10.12688/f1000research.14819.2)
Supplement: Supplementary file 3 [file f1000research-7-17614-s0002.tgz › b2a79496-4ada-47c4-9682-a99ce867836d.zip!/Eight Iranian Health Journals/The Journal of Toloo-e-Behdasht_12.docx]

© The Journal of Toloo-e-Behdasht. This work is licensed under CC BY 4.0 (https://creativecommons.org/licenses/by/4.0/).

Volume & Issue: دوره 15 - شماره 2

Title: پیوند بهداشت و عبادات در قرآن و روایات

http://tbj.ssu.ac.ir/article-1-2190-fa.html

چکیده در اسلام پاکیزگی پیش شرط پذیرش بسیاری از عبادات به شمار رفته است. در بسیاری موارد نیز پاکیزگی به عنوان یک عمل عبادی و مستحب قلمداد شده است. این پاکیزگی و طهارت را می‌توان از دو جنبه فردی و اجتماعی مورد بررسی قرار داد. بنابراین می‌توان از پیوند عبادات و بهداشت فردی و اجتماعی سخن گفت. به گونه ای که بر اساس قرآن و روایات می‌توان یکی از مأموریت‌های پیامبر (ص) بهداشت و پاکیزگی و نظافت لباس، بدن، خوراک و سایر ابعاد زندگی انسان بوده است. تا جایی که اسلام در حد یک قاعده کلی، نظافت را از ایمان می‌داند. این موضوع در ارتباط با نماز، غسل و ... کاملاً مشهود است. در این مقاله برآنیم تا با استمداد از قرآن و روایات پیوند بهداشت فردی و اجتماعی با عبادات را جویا شویم. می‌توان گفت، در قرآن مقصود از این مفاهیم بهداشت ظاهری و باطنی است. برخی آیات بیان کرده اند که انسان از غذاهای سالم استفاده شود و یا اینکه از مومنین خواسته هنگام مستی به نماز نزدیک نشوند و نیز از خوردن گوشت مردار بپرهیزند، چراکه مردار غذای ناسالم و غیر بهداشتی به شمار می رود. در روایات اسلامی تاکید فراونی بر نظافت و ارتباط آن با ایمان شده است. مثلا جمله مشهور پیامبر اکرم (ص) که می فرماید: «نظافت (و بهداشت) قسمتی از ایمان است»؛ این نشان می دهد که ایمان انسان بدون رعایت مسایل بهداشتی کامل نمی شود. توجه به انواع غسل‌های واجب واستحبابی ووضونیزدرزندگی عبادی، اهمیت نظافت رادراسلام نشان می‌دهد. پس نظافت عنصر اساسی بهداشت است و بدون رعایت آن نمی‌توان از سلامتی برخورداربود. از این رو، یک فرد نمی‌تواند درعین مؤمن بودن، به اصول نظافت بی اعتنایی کند.

Title: مقایسه مدل‌های آمیخته خطی تعمیم‌یافته و مدل‌های خطی تعمیم‌یافته در تعیین عوامل مرتبط با بیماری دیابت نوع2 در استان یزد

http://tbj.ssu.ac.ir/article-1-2191-fa.html

مقدمه: بیماری دیابت از جمله بیماری‌های مزمنی است که شیوع آن بسیار زیاد و روز به روز درحال افزایش است. در این مطالعه ضمن تعیین عوامل موثر بر بیماری دیابت به مقایسه دو مدل خطی تعمیم‌یافته وخطی آمیخته تعمیم‌یافته می‌پردازیم. روش بررسی: داده‌های این مطالعه مربوط به طرح تحقیقاتی بررسی شاخص‌های اپیدمیولوژیک بیماری دیابت بزرگسالان در گروه سنی 30سال وبالاتر شهری استان یزد می‌باشد. در این مطالعه جمعا 2795 نفر با انجام آزمایش قند خون از لحاظ ابتلا به دیابت بررسی شدند. برای تحلیل داده‌ها با استفاده از مدل رگرسیون لجستیک آمیخته ورگرسیون لجستیک معمولی از نرم افزار  R استفاده شد. یافته‌ها: در این مطالعه متغیر‌های سابقه خانوادگی ابتلا به دیابت، سن، شاخص توده بدنی و دورکمر به دور باسن در هردو مدل معنی‌دار شد ( 001/p<.) ، با این تفاوت متغیر که شغل در مدل رگرسیون لجستیک معمولی در سطح1/0 معنی‌دار بود ولی در مدل رگرسیون لجستیک آمیخته معنی دار نبود. همچنین، متغیرهای  مساحت خانه، سطح تحصیلات و جنسیت در هیچ یک معنی دار نشد. با توجه به مقادیر نسبت شانس نیز در برخی از آنها شاهد تفاوت قابل‌توجهی بین دو مدل هستیم. با توجه به خطای استاندارد ضرایب ومقایسه مقادیر آن در دو مدل، شاهد کم برآوردی در مدل رگرسیون لجستیک معمولی بودیم. نتیجه‌گیری: بکار‌گیری مدل‌های آمیخته تعمیم‌یافته منجر به نتایج دقیق‌تر می‌شود و ازکم برآوردی خطای استاندارد ضرایب جلوگیری می‌کند.

Title: بررسی میزان حذف سورفاکتانت‌های آنیونی و COD فاضلاب کارواش با فرایند ازن زنی در یک راکتور با بستر آکنده

http://tbj.ssu.ac.ir/article-1-2192-fa.html

مقدمه: کارواش یکی از صنایعی است که میزان زیادی آب مصرف کرده و فاضلاب آنها نیز حاوی انواع آلاینده‌‌ها ) دترجنت‌ها، روغن و چربی، فلزات و ...) می‌باشد. تصفیه فاضلاب ناشی از این صنعت می تواند کمک بزرگی به حفظ منابع آب سطحی و زیرزمینی نماید. بنابراین در مطالعه حاضر کاربرد فرایند ازن زنی در تصفیه فاضلاب حاصل از شستشوی اتومبیل در یکی از کارواش‌های شهر یزد مورد بررسی قرار گرفت. روش بررسی: این مطالعه در مقیاس آزمایشگاهی و به صورت پایلوت انجام شد. نمونه‌ها به صورت روزانه از یک کارواش جمع‌آوری گردید. با استفاده از یک راکتور استوانه‌ای شکل (با ارتفاع 1 متر و قطر داخلی 5 سانتی‌‌متر) حاوی بستر پلاستیکی، درصد حذف پارامترهایCOD با روش هضم و سورفکتانت آنیونی با روش استاندارد استخراج کلروفرم در طی فرآیند ازن زنی در دز ازن 7/0 و 1/1 میلی‌گرم در دقیقه در زمان‌های واکنش 30، 60 و90 دقیقه و pH 7، 9 و11 تعیین شد. یافته‌ها: نتایج نشان داد راندمان حذف COD و سورفکتانت‌های آنیونی با افزایش pH و زمان تماس افزایش می‌یابد به طوری که در 11=pH و بعد از زمان واکنش 90 دقیقه برای دز ازن 1/1 میلی‌گرم در دقیقه به ترتیب راندمان حذف 57/68 و 71/77 درصد بدست آمد.  نتیجه‌گیری: نتایج این مطالعه نشان داد که فرایند ازن‌زنی در یک راکتور با بستر آکنده دارای توانایی قابل‌توجهی در حذف COD و سورفکتانت‌های آنیونی می‌باشد. اما این فرایند به تنهایی قادر به حذف محتوای آلی فاضلاب کارواش نبوده و در صورت نیاز می‌توان از آن بصورت ترکیب با سایر روش‌ها جهت تصفیه فاضلاب کارواش‌ها استفاده نمود.

Title: بررسی رابطه رضایت از زندگی و سلامت اجتماعی زنان شهر یزد

http://tbj.ssu.ac.ir/article-1-2193-fa.html

مقدمه: ضرورت توجه به سلامت اجتماعی زنان یک امر بدیهی و آشکار بوده و به همان میزان که ﺳﻼﻣﺖ ﺧـﺎﻧﻮاده، ﺟﺎﻣﻌـﻪ و ﻣﺤـﻴﻂ زﻳﺴـﺖ ضروری است، تأمین سلامت اجتماعی زنان نیز امری اجتناب‌ناپذیر است. از سوی دیگر رضایت از زندگی بر عواملی که پیش‌بینی‌کننده سلامتی هستند تأثیر‌گذار بوده و در مقابل ارتباط بین نارضایتی و سایر ریسک فاکتورهای سلامتی مورد تأیید قرار گرفته است. بر این اساس پژوهش حاضر می‌کوشد تا وضعیت سلامت اجتماعی زنان شهر یزد و رابطه آن را با رضایت از زندگی مورد بررسی قرار دهد. روش بررسی: تحقیق حاضر یک مطالعه مقطعی به شیوه پیمایشی است. جامعه آماری تحقیق شامل کلیۀ زنان 60-18 ساله شهر یزد در سال 1393 است. حجم نمونه براساس فرمول کوکران 288 نفر تعیین شد و با استفاده از روش نمونه‌گیری خوشه‌ای متناسب (PPS) انجام گرفت. داده‌ها بر اساس پرسشنامه استاندارد سلامت اجتماعی کییز (1998) و رضایت از زندگی داینر و همکاران (1985) جمع‌آوری شدند. یافته‌ها: میزان سلامت اجتماعی و رضایت از زندگی پاسخگویان در سطح متوسط بود. ضریب همبستگی پیرسون بین میزان رضایت از زندگی و ابعاد سلامت اجتماعی نیز عبارت است از: انسجام اجتماعی (215/0r=)، مشارکت اجتماعی (176/0r=)، ﭘﺬﻳﺮﺵ ﺍﺟﺘﻤﺎﻋﻲ (311/0r=)، شکوفایی اجتماعی (175/0r=) و همبستگی اجتماعی (213/0r=) که به لحاظ آماری معنادار هستند. علاوه بر این نتایج مدل‌سازی معادلات ساختاری نیز نشان داد که رابطه معناداری بین رضایت از زندگی و سلامت اجتماعی  زنان وجود دارد. نتیجه‌گیری: نتایج نشان می‌دهد که بین میزان رضایت از زندگی و سلامت اجتماعی رابطه معنی‌دار و مستقیمی وجود دارد و با افزایش رضایت از زندگی، سلامت اجتماعی نیز افزایش پیدا می‌کند.

Title: بررسی عوامل مرتبط با رفتارهای پیشگیری‌کننده از پوکی استخوان براساس الگوی اعتقاد بهداشتی (HBM) در زنان 49-20 ساله شهر یزد

http://tbj.ssu.ac.ir/article-1-2194-fa.html

مقدمه: بیماری پوکی استخوان یکی از شایع‌ترین مشکلات سیستم عضلانی- اسکلتی و نوعی اختلال متابولیکی استخوان می‌باشد که معمولاً بدون علامت بوده و در صورت عدم پیشگیری و درمان، اولین بار خود را با شکستگی استخوان نشان می­دهد. هدف از این مطالعه، بررسی عوامل اجتماعی مرتبط با رفتارهای پیشگیری‌کننده از پوکی استخوان در زنان بر اساس مدل اعتقاد بهداشتی می‌باشد. روش بررسی: این پژوهش یک مطالعه توصیفی است که به صورت مقطعی انجام گردید. جامعه مورد بررسی شامل 278 نفر از افراد مراجعه‌کننده به مراکز بهداشتی-درمانی وابسته به دانشگاه علوم پزشکی شهید صدوقی یزد بودند. گردآوری اطلاعات توسط پرسشنامه و به صورت خودگزارش‌دهی تکمیل گردید. پایایی و روایی پرسشنامه مورد سنجش قرار گرفت و تایید گردید. داده­ها با استفاده از نرم افزار SPSS نسخه 16 مورد تجزیه و تحلیل قرار گرفتند و در انجام آن از آزمون­های آماری کروسکال والیس همراه با آزمون تعقیبی دان، من ویتنی، ضریب همبستگی اسپیرمن، تحلیل رگرسیون استفاده شد. یافته‌ها: در میان رفتارهای پیشگیری‌کننده از پوکی استخوان بیشترین فراوانی مربوط به عدم مصرف سیگار یا در معرض آن قرار گرفتن و کمترین فراوانی مربوط به انجام فعالیت بدنی منظم در طول هفته می­باشد. بر اساس ضریب همبستگی اسپیرمن، میزان آگاهی، منافع درک شده، راهنما برای عمل و خودکارآمدی با عملکرد زنان در خصوص رفتارهای پیشگیری‌کننده از پوکی استخوان همبستگی مثبت و مستقیم و موانع درک شده با عملکرد زنان همبستگی معکوس و معنی‌داری داشت (001/0P<). در مجموع متغیرهای الگو 36% از واریانس رفتارهای پیشگیری‌کننده را پیش‌بینی نمودند و از میان این متغیرها سازه خودکارآمدی قوی‌ترین پیش‌بینی‌کننده رفتار بود (000/0P=). نتیجه‌گیری: با توجه به نتایج به دست آمده، ارائه برنامه‌های آموزشی مناسب با تأکید بر افزایش آگاهی و همچنین بالا بردن سطح سازه‌های مدل اعتقاد بهداشتی و کاهش موانع در زمینه بیماری‌هایی چون پوکی استخوان توسط کادر مراکز بهداشتی درمانی پیشنهاد می­شود.

Title: کاربرد تئوری رفتار برنامه‌ریزی شده در پیشگویی وابستگی به نیکوتین در زنان مصرف‌کننده قلیان شهر بوشهر سال 93-1392

http://tbj.ssu.ac.ir/article-1-2196-fa.html

چکیده مقدمه: امروزه اپیدمی جهانی تنباکو منجر به مرگ میلیون‌ها نفر در سال می‌شود. مطالعه حاضر جهت شناسایی عوامل پیشگویی‌کننده وابستگی به نیکوتین در زنان مصرف‌کننده قلیان در شهر بوشهر طراحی شده است. روش‌بررسی: پژوهش حاضر مطالعه­ای توصیفی- تحلیلی از نوع مقطعی (cross-sectional) می‌باشد که در آن تعداد 430 زن مصرف‌کننده قلیان ساکن شهر بوشهر مورد بررسی قرار گرفتند. به منظور طراحی پرسشنامه محقق ساخته با تعداد 20 نفر از زنان قلیانی مصاحبه ساختارمند انجام و سپس پرسشنامه طراحی گردید. جمع‌آوری داده‌ها به روش نمونه‌گیری در دسترس و گلوله برفی بود. پس از جمع‌آوری اطلاعات، تجزیه و تحلیل داده‌ها توسط نرم‌افزار آماری SPSS و با استفاده از آزمون‌های آماری مناسب انجام شد. یافته‌ها: در این مطالعه میانگین سنی نمونه‌ها 57/13± 73/36 سال بود. میانگین و انحراف‌معیار نمره کلی رفتار وابستگی به نیکوتین 63/12± 71/40 بود. بالاترین نمره، مربوط به رفتار وابستگی به نیکوتین و کمترین نمره در سازه کنترل رفتاری درک شده بدست آمد. کلیه سازه‌ها بجز نگرش ابزاری و هنجار ذهنی، تبیین‌کننده رفتار وابستگی به قلیان بوده و از این بین خودکارآمدی بیشترین و نگرش احساسی کمترین تبیین‌کنندگی را داشتند (ضریب تبیین 30%). نتیجه‌گیری: با توجه به وضعیت نامطلوب رفتار وابستگی به نیکوتین در زنان مصرف‌کننده قلیان، برنامه‌های مداخلاتی جهت ارتقاء خودکارآمدی افراد در کاهش این رفتار، کاهش احساسات مطلوب نسبت به قلیان و رفع هنجار توصیفی توصیه می‌گردد.

Title: تأثیرطراحی و ارزشیابی برنامه آموزش بهداشت بر میزان آگاهی، نگرش و عملکرد خانواده بیماران مسلولین شهرستان زابل در ارتباط با بیماری سل

http://tbj.ssu.ac.ir/article-1-2197-fa.html

چکیده مقدمه: سل یکی از قدیمی‌ترین بیماری‌های بشر است و در طول سالیان دراز همواره به‌عنوان یکی از عوامل مرگ‌ومیر و ناتوانی انسان‌ها مطرح بوده است. ازآنجاکه سل معلول فقر وفقر معلول جهل وجهل مهم‌ترین شاخص ضعف فرهنگی است، هدف این مطالعه طراحی وارزشیابی برنامه آموزش بهداشت در خانواده مسلولین شهر زابل نسبت به بیماری سل بود. روش بررسی: نوع مطالعه مداخله‌ای- نیمه تجربی (قبل و بعد) بود.جامعه مورد مطالعه شامل تمام افراد خانواده‌های مسلولین شهرستان زابل ( 95 نفر) بودند. ابزار جمع‌آوری اطلاعات در این پژوهش، پرسشنامه طراحی‌شده در زمینه آگاهی، نگرش و عملکرد در رابطه با بیماری سل بود که مشتمل بر 43 سؤال بود.آموزش از طریق سخنرانی وآموزش چهره به چهره انجام شد. اطلاعات با استفاده از نرم‌افزار 18SPSS و با استفاده از آمار توصیفی و تحلیلی و همچنین استفاده از آزمون‌های آماری Tزوج وTمستقل و آنالیز واریانس یک‌طرفه و سپس آزمون توکی، اطلاعات مورد تجزیه‌وتحلیل قرار گرفت و05/0 P< به‌عنوان سطح معنی‌داری در نظر گرفته شد. نتایج: میانگین آگاهی افراد موردمطالعه در قبل از آموزش 93/9 (از مجموع 20 امتیاز) بود که بعد از آموزش به 42/17 افزایش پیدا کرد و میانگین نمره نگرش افراد موردمطالعه در قبل از آموزش 67/3(از مجموع 9 امتیاز) بود که بعد از آموزشبه 45/7 افزایش پیدا کرد و میانگین نمره عملکرد افراد موردمطالعه در قبل از آموزش 04/7(از مجموع 14 امتیاز) بود که بعد از آموزشبه 77/12 افزایش پیدا کرد و این اختلاف در سطح 05/0≥p معنی‌دار بود. نتیجه‌گیری: برنامه آموزشی مؤثر می‌تواند سطح آگاهی، نگرش و عملکرد افراد خانواده را در مورد بیماری‌های مختلف افزایش داده و درنتیجه منجر به ارتقای سلامتی جامعه گردد.لذا مسئولان و برنامه ریزان بهداشتی کشور می‌توانند با برگزاری کلاس‌های آموزشی و ارتقای سطح آگاهی جامعه گام مؤثری در جهت بهبود وضعیت سلامت جامعه بردارند.

Title: بررسی عوامل مرتبط با خود مراقبتی در بیماران مبتلا به مولتیپل اسکلروزیس( MS) شهر یزد بر اساس مدل اعتقاد بهداشتی

http://tbj.ssu.ac.ir/article-1-2198-fa.html

مقدمه: خود‌مراقبتی یکی از جنبه‌های مهم درمان در بیماران مولتیپل اسکلروزیس است. نظر به لزوم توجه به نیازها، حقایق و تعیین‌کننده‌های یک رفتار و جهت به حداکثر رساندن تاثیر برنامه‌های آموزشی، مطالعه حاضر با هدف تعیین خودمراقبتی بیماران مبتلا به مولتیپل اسکلروزیس و عوامل مرتبط با آنها، بر اساس الگوی اعتقاد بهداشتی انجام گرفت. روش بررسی: پژوهش حاضر یک مطالعه توصیفی از نوع همبستگی بود. 120 بیمار به روش نمونه‌گیری تصادفی ساده از بین بیماران عضو انجمن MS شهر یزد وارد مطالعه شدند. ابزار جمع‌آوری اطلاعات، پرسشنامه‌ای بود که عوامل مرتبط با رفتارهای خودمراقبتی در بیماری MS مشتمل بر درک بیماران از علایم و سازه‌های مدل اعتقاد بهداشتی را همراه با رفتارهای خودمراقبتی ارزیابی می‌نمود. داده‌ها با استفاده از آزمون‌های همبستگی پیرسون، آنالیز رگرسیون چندگانه، t مستقل، همبستگی اسپیرمن و آنالیز واریانس یک طرفه در نرم افزار spss نسخه 20 تجزیه و تحلیل شد. یافته‌ها: آزمودنی‌ها به طور متوسط 47/51%از رفتار‌های خودمراقبتی را انجام می‌دادند و در میان حوزه‌های مختلف رفتارهای خود‌مراقبتی، استفاده از وزنه‌های کوچک برای کاهش ناهماهنگی حرکتی و لرزش دست‌ها و پاها‌، اختصاص زمان منظمی برای دفع ادرار و انجام ورزش‌های مناسب دارای کمترین فراوانی بود قدرت پیش‌بینی‌کنندگی شدت درک‌شده و راهنما برای عمل در پیش‌بینی رفتارهای خودمراقبتی معنی‌دار بود که در این میان شدت درک‌شده پیشگویی‌کننده قوی‌تر بود. در ضمن کل سازه‌های مدل اعتقاد بهداشتی 8/9%تغییرات رفتارهای خودمراقبتی را پیش‌بینی می‌کنند. نتیجه گیری: با توجه به اینکه مهمترین عوامل مرتبط با خودمراقبتی شدت درک شده و راهنما برای عمل مشخص گردید. بنابراین لازم است در برنامه‌های مداخله‌ای بر موارد فوق تاکید بیشتری شود. از سوی دیگر با توجه به قدرت پیش‌بینی‌کنندگی پایین مدل اعتقاد بهداشتی پیشنهاد می‌شود در مطالعات بعدی از مدل‌های دیگر اس

Title: بررسی ارتباط کم‌خونی با عوامل دموگرافیک در کودکان 12-7 ساله یزدی

http://tbj.ssu.ac.ir/article-1-2200-fa.html

مقدمه: کم‌خونی یکی از مشکلات بهداشتی عمومی در سراسر جهان و از جمله درکودکان می‌باشد. لذا مطالعه حاضر که در پی یافتن عوامل مرتبط با کم‌خونی می‌باشد با هدف بررسی ارتباط کم‌خونی با عوامل دموگرافیک در کودکان 12-7 ساله یزدی انجام پذیرفت. روش بررسی: در این مطالعه مقطعی 390 نفر  از دانش‌آموزان 12-7 ساله مدارس ابتدایی شهرستان یزد به روش نمونه‌گیری خوشه ای چند مرحله‌ای به طور تصادفی انتخاب شدند. تنها دانش‌آموزان سالم وارد این مطالعه شدند به علاوه در گروه دختران افرادی که سیکل‌های ماهانه آنها شروع شده بود وارد مطالعه نشدند. اطلاعات فردی و دموگرافیک با استفاده از پرسشنامه جمع‌آوری گردید. قد و وزن افراد به روش استاندارد اندازه‌گیری شد. هموگلوبین سرم کمتر از 5/11 گرم در دسی‌لیتر کم‌خونی، هموگلوبین 5/13-5/11 گرم در دسی‌لیتر در معرض خطر کم‌خونی و فریتین کمتر از 15 میکروگرم در دسی لیتر کم‌خونی شدید یا  فقر آهن تعریف شد. یافته‌ها: کم‌خونی در3/1% کودکان مورد مطالعه مشاهده شد، اما 5/51% کودکان در معرض خطر کم‌خونی بودند. 1/9% کودکان کم‌خونی شدید داشتند. در بین عوامل دموگرافیک مورد بررسی سن و جنسیت ارتباط آماری معنی‌داری با کم‌خونی داشت. به طوری که کودکان 8-7 ساله و دختران دارای شیوع بالاتری از کم‌خونی بودند. نتیجه‌گیری: بیش از نیمی از نوجوانان یزدی (8/52% ) دارای کم‌خونی یا در معرض کم‌خونی فقر آهن می‌باشند. بالا بودن بیشترین شیوع کم‌خونی در دختران قبل از شروع سیکل‌های ماهانه بسیار حائز اهمیت می‌باشد.

Title: بررسی تاثیر مشاوره قبل از ازدواج بر آگاهی و نگرش زوجین در شهر یزد

http://tbj.ssu.ac.ir/article-1-2199-fa.html

چکیده مقدمه: برگزاری صحیح کلاسهای آموزشی مشاوره قبل از ازدواج به زوجین کمک می‌کند تا آگاهی لازم در زمینه مسائل مختلف بهداشت باروری را کسب نمایند. هدف از این مطالعه، تعیین تاثیر مشاوره قبل از ازدواج بر آگاهی و نگرش زوجین در شهر یزد بوده است. روش بررسی: این مطالعه نیمه تجربی و از نوع قبل و بعد بوده که در آن 200 نفر از زوجین شرکت‌کننده در کلاسهای مشاوره قبل از ازدواج به صورت تصادفی انتخاب شدند. جمع‌آوری اطلاعات توسط پرسشنامه بوده که در دو مرحله ی قبل و بعد از آموزش توزیع و پس از تکمیل جمع‌آوری گردید. در نهایت داده ها توسط نرم افزار SPSS نسخه 18 و آزمون‌های آماری t-test و ANOVA مورد تجزیه و تحلیل قرار گرفتند. یافته‌ها: داده‌ها نشان داد که زوجین قبل از شرکت درکلاس‌های مشاوره به ترتیب 6/37 و 1/48 درصد و بعد از آموزش به ترتیب 1/65 و 6/57 درصد از نمره مربوط به آگاهی و نگرش را کسب کرده اند. همچنین میانگین نمره آگاهی و نگرش بر حسب جنس، سطح تحصیلات و شغل از لحاظ آماری معنی‌دار بوده است(05/0P≤). نتیجه‌گیری: با توجه به تغییر کم نگرش زوجین، پیشنهاد می‌شود بعد از کلاس‌های مشاوره فرصتی به زوجین داده شود تا سوالات خود را به صورت خصوصی بیان کنند. همچنین برای رسیدن به تغییر رفتار نسبتا پایدار در زوجین جوان و ارتقای سطح سلامت آنان، باید در کنار برگزاری کلاس‌های آموزشی پیش از ازدواج به کیفیت این کلاس‌ها نیز توجه بیشتری شود.

Title: بررسی کارایی فرآیند انعقاد الکتریکی در حذف کدورت و جامدات معلق حاصل از پساب شستشوی صافی شنی تند

http://tbj.ssu.ac.ir/article-1-2202-fa.html

چکیده مقدمه: با روش­های مناسب می­توان بیش از 95 درصد پساب شستشوی معکوس فیلتر را بازیافت نمود. هدف از انجام این مطالعه بررسی کارایی فرایند انعقاد الکتریکی در حذف کدورت و جامدات معلق از پساب شستشوی صافی شنی تند تصفیه خانه آب شماره یک کرج می‌باشد. روش بررسی: این مطالعه به صورت تجربی در مقیاس آزمایشگاهی و به صورت پایلوت در سیستم ناپیوسته انجام شد. مخزنی با جنس پلاکسی گلاس با حجم 4 لیتر، حاوی 4 عدد الکترود صفحه‌ای از جنس آلومینیوم و آهن به یک منبع تغذیه جریان مستقیم متصل شد. نمونه­ها هر 15 دقیقه از میانه راکتور جهت اندازه‌گیری کدورت و جامدات معلق جمع‌آوری و مورد آزمایش قرار گرفتند. تأثیر پارامتر­های مختلفی از قبیل دانسیته جریان، زمان واکنش و پتانسیل الکتریکی مورد مطالعه قرار گرفت. تعداد کل نمونه­های مورد آزمایش 48 نمونه بود. کدورت به روش نفلومتری و کل مواد جامد معلق  به  روش وزن سنجی سنجش شد. یافته­ها: بیشترین میزان حذف کدورت و جامدات معلق در زمان واکنش 60 دقیقه، دانسیته جریان mA/cm210 و اختلاف پتانسیل 45 ولت مشاهده شد. بیشترین راندمان حذف کدورت در الکترود آلومینیومی و آهنی به ترتیب برابر با 83/96 درصد و 77/83 درصد بود. همچنین بیشترین میزان حذف جامدات معلق در الکترود آلومینیومی و آهنی به ترتیب برابر با 73/96 درصد و 22/86 درصد بود. نتیجه­گیری: نتایج این مطالعه نشان داد که فرآیند انعقاد الکتریکی می‌تواند گزینه مناسبی برای حذف کدورت و جامدات معلق از پساب شستشوی صافی شنی تند باشد. کارایی الکترود آلومینیومی در حذف کدورت و جامدات معلق بیشتر از الکترود آهنی بود.

Title: بررسی آگاهی و نگرش افراد مبتلا به پریودنتیت در مورد اثر سیگار بر سلامت پریودنتال

http://tbj.ssu.ac.ir/article-1-2201-fa.html

مقدمه: گرچه عامل اصلی ایجادکننده پریودنتیت، پلاک میکروبی است ولی نقش سیگار به عنوان یک عامل خطر مهم در فرآیند پیشرفت بیماری‌های پریودنتال، به اثبات رسیده است. هدف از این مطالعه بررسی میزان آگاهی و نگرش افراد مبتلا به پریودنتیت در مورد اثر سیگار بر سلامت پریودونتال بود. روش بررسی: در این مطالعه توصیفی- مقطعی، 300 بیمار مبتلا به پریودنتیت با دامنه سنی 74-18 سال به روش نمونه‌گیری آسان از مراجعه‌کنندگان به بخش پریودنتولوژی دانشکده دندانپزشکی شهید صدوقی و کلینیک تخصصی خاتم الانبیاء شهر یزد انتخاب شدند. داده‌ها با استفاده از پرسشنامه جمع‌آوری شد و توسط نرم‌افزار آماری SPSS نسخه 17 و آزمون‌های آماری chi-square،t-test و ANOVA مورد تجزیه و تحلیل قرار گرفت. یافته‌ها: میانگین نمره کلی آگاهی بیماران از اثرات سیگار بر سلامت پریودنتال 4/18±7/86 و نمره نگرش آن‌ها 3/7±1/48 بود. ارتباط معنی‌داری میان آگاهی و نگرش بیماران با سطح تحصیلات آن‌ها وجود داشت. در این مطالعه 42٪ افراد سیگاری حداقل برای یک بار اقدام به ترک سیگار کرده بودند که تنها 3/14% از آنها به دلیل مسائل مربوط به سلامت دهان و دندان و مشورت با دندانپزشک بوده است. نتیجه‌گیری: نتایج پژوهش نشان داد که اگرچه میزان آگاهی بیماران از اثرات سیگار بر سلامت پریودنتال خوب است ولی نگرش آن‌ها در این باره متوسط ارزیابی شد. شاید علت اصلی این‌که بیماران با داشتن آگاهی نسبتاً خوب نسبت به اثرات زیانبار سیگار بر سلامت پریودنتال باز به کشیدن سیگار ادامه می‌دهند، همین نگرش متوسط به این موضوع باشد. این مطالعه نشان داد دندانپزشکان نقش کمی در آگاه کردن بیماران به عهده داشتند.

Title: بررسی اثر یک دوره تمرین مقاومتی بر برخی عوامل خطر زای قلبی عروقی در زنان مبتلا به سندرم متابولیک

http://tbj.ssu.ac.ir/article-1-2203-fa.html

مقدمه: سندرم متابولیک به‌عنوان یک عامل خطرزابرای بیماری‌های مزمن متعددی مانند دیابت نوع2 و بیماری قلبی- عروقی محسوب می‌شود که از طریق برخی عوامل مانند تغذیه ناسالم، روش زندگی بی‌تحرک و استعداد ژنتیکی ایجاد می‌گردد. در حالی که سطوح بالاتری از قدرت عضلانی با خطر پایین‌تر سندرم متابولیک همراه می‌باشد. لذا هدف از تحقیق حاضر بررسی پاسخ برخی عوامل خطرزای قلبی- عروقی در زنان مبتلا به سندرم متابولیک به یک دوره تمرین مقاومتی است. روش بررسی: 26 زن یائسه سالم غیرفعال به‌صورت داوطلبانه هدفمند جهت شرکت در پژوهش انتخاب و به‌طور تصادفی به دو گروه تجربی و گواه تقسیم شدند.آزمودنی‌ها در آزمون‌های تن‌سنجی شامل اندازه‌گیری قد، دور کمر و لگن، وزن، چربی زیر‌جلدی و نمونه‌گیری خونی شرکت کردند. گروه تجربی تمرینات مقاومتی را به مدت 10 هفته و هر هفته 3 جلسه با 40 تا 50 درصد یک تکرار بیشینه انجام دادند. یافته‌ها : نتایج نشان دادکه پس از 10 هفته تمرین مقاومتی در گروه تجربی شاخص‌های وزن                   (001/0>P )،کلسترول تام(03/0>P) وتری‌گلیسرید(001/0>P) نسبت به گروه کنترل کاهش معنی‌دار داشت. همچنین در گروه تجربی نسبت به پیش‌آزمون تغییرات معنی‌داری در میانگین نمایه توده بدن، اندازه دور کمر، درصد چربی، فشار خون سیستولیک و کلسترول-HDL مشاهده گردید، اگرچه این تغییرات در بین دو گروه معنی‌دار نبود. نتیجه‌گیری: در مجموع یافته‌های پژوهش نشان داد که یک برنامه فعالیت ورزشی مقاومتی می‌تواند باعث بهبود عوامل خطرزای قلبی عروقی در زنان مبتلا به سندرم متابولیک شود. البته ساز و کارهای مؤثر در بهبود علائم سندرم متابولیک متعاقب فعالیت‌های ورزشی به‌وضوح مشخص نشده است.

Title: بررسی توانایی نانوذرات سلولز کونژوگه شده با پلی‌گوانین در جذب سم پاتولین در آب سیب‌های آلوده

http://tbj.ssu.ac.ir/article-1-2204-fa.html

چکیده مقدمه: پاتولین سمی خطرناک است که به‌وسیله انواع مختلفی از کپک‌ها تولید می‌شود. هدف از این مطالعه بررسی توانایی نانوذرات سلولز کونژوگه شده با پلی گوانین در جذب سم پاتولین در آب سیب‌های آلوده بود. روش­ بررسی: ابتدا نانوذرات سلولز سنتز و سپس با کمک کراس لینکر به مولکول پلی‌گوانین متصل گردید. سپس سریال غلظت پاتولین(1000 و 500 و 250 و 125 میکروگرم بر لیتر) در آب سیب تهیه و به همه آنها نانوذرات کونژوگه اضافه شد و همگی در 37 درجه سانتی‌گراد انکوبه گردیدند. بعد از انکوباسیون، غلظت پاتولین در هر لوله با اسپکتروسکوپی در طول موج 340 نانومتر به‌دست آمده و نهایتاً درصد جذب در هر لوله حساب شد. برای شبیه‌سازی مولکولی نیز ساختار‌های اولیه پاتولین و نانوذرات کونژوگه شده با پلی‌گوانین در برنامه Hyper Chem وارد و به‌مدت 50 پیکو‌ثانیه انرژی بین مولکولی این دو ساختار محاسبه شد. یافته‌ها: این مطالعه نشان داد که رابطه معنی‌داری بین غلظت اولیه پاتولین و میزان جذب سم توسط نانوذرات وجود دارد(05/0P=). حداکثر جذب نیز حدود هفتاد درصد گزارش گردید. همچنین شبیه‌سازی رایانه‌ای نیز جذب را تایید نمود. نتیجه‌گیری: از این مطالعه می‌توان نتیجه گرفت که نانوذرات کونژوگه شده با پلی‌گوانین جاذب مناسبی برای سم پاتولین بوده و باید در مطالعات آینده به لحاظ کاربردی نیز مورد استفاده قرار گیرد.

Title: بررسی اثربخشی آموزش خوددلگرم‌سازی شوانکر بر میزان تاب‌آوری و خوش‌بینی مادران دارای کودکان معلول‌ذهنی

http://tbj.ssu.ac.ir/article-1-2206-fa.html

مقدمه: یک‌کودک کم‌توان ذهنی، به عنوان یک فشار خردکننده روی والدین‌می‌باشد. در نتیجه آموزش مادران این کودکان از اهمیت بسزایی برخوردار است. یکی از فعالیت‌های مفید در این زمینه آموزش خوددلگرم‌سازی می‌باشد. بدین منظور مطالعه حاضر با هدف بررسی اثربخشی خوددلگرم‌سازی بر میزان تاب‌آوری و خوش‌بینی مادران دارای کودک کم‌توان ذهنی انجام گرفت. روش بررسی: به شیوه‌ نیمه آزمایشی پیش آزمون- پس آزمون با گروه کنترل اجرا گردید. گروه نمونه شامل 24 نفر از مادران دارای کودک کم‌توان ذهنی یزد در سال 94-1393بودند، که به صورت داوطلبانه (غیرتصادفی) انتخاب گردیدند و در نهایت به صورت تصادفی در دو گروه آزمایش و کنترل (در هر گروه 12نفر) جایگزین شدند. میزان تاب‌آوری و خوش‌بینی شرکت‌کنندگان در این پژوهش به ترتیب توسط پرسشنامه‌ تاب‌آوری کانر- دیویدسون (CD-RISC) و جهت‌گیری زندگی(LOT)  در پیش آزمون و پس آزمون سنجیده شد و گروه آزمایش در 10 جلسه‌ 1:30 ساعته آموزش خوددلگرم سازی طی 10 هفته متمادی در روزهای یکشنبه در آموزشگاه تلاش یزد شرکت نمودند. برای تجزیه و تحلیل داده‌ها از آزمون تحلیل کوورایانس چند متغیری (MANCOVA)، تحلیل کوورایانس یک متغیری (ANCOVA) با استفاده از نرم افزار SPSS نسخه22 استفاده شد. یافته‌ها: تحلیل کوواریانس بر روی داده‌ها نشان داد که جلسات آموزشی تاثیر معنی‌داری بر تاب‌آوری و خوش‌بینی گروه آزمایش داشته است(05/0P<). نتیجه‌گیری: آموزش خوددلگرم‌سازی می‌تواند به احتمال زیاد افزایش تاب‌آوری و خوش‌بینی را در پی داشته باشد.

Title: بررسی رابطه بین کیفیت زندگی و امید به زندگی در بیماران مبتلا به سرطان پستان

http://tbj.ssu.ac.ir/article-1-2205-fa.html

چکیده مقدمه: از جمله اختلالاتی که به شدت بر سلامت و در نتیجه کیفیت زندگی افراد تأثیر می گذارد بیماری های مزمن نظیر سرطان است که پس از سرطان پوست دومین سرطان شایع در زنان می‌باشد. با توجه به رشد روز افزون سرطان پستان،  پژوهش حاضر با هدف بررسی رابطه بین کیفیت زندگی و امید به زندگی در بیماران مبتلا به سرطان پستان صورت گرفت.   روش بررسی: این مطالعه به روش توصیفی-مقطعی انجام شد و جامعه مورد بررسی 80 نفر از بیماران مبتلا به سرطان پستان یکی از مراکز شیمی درمانی شهر یزد بودند.ابزار جمع آوری اطلاعات در این بررسی پرسشنامه کیفیت زندگی(SF-36) و پرسشنامه امیدواری اسنایدر بود که به روش مصاحبه با بیماران تکمیل گردید. داده‌ها با استفاده ازspss ویرایش 18 وآزمون‌های آماریt-test وضریب همبستگی پیرسون و رگرسیون مورد تجزیه و تحلیل آماری قرار گرفت. نتایج: یافته‌ها نشان داد که با استفاده از آزمون ضریب همبستگی پیرسون و با اطمینان 95% ، 288/0r= و01/0p= می‌توان گفت که همبستگی مثبت و معنی داری بین کیفیت زندگی و امید به زندگی وجود دارد. میانگین امید به زندگی در این بیماران67/3± 63/23 و میانگین کیفیت زندگی آنها6/19 ا± 32/47 می‌باشد. نتیجه‌گیری: بیمارانی که از کیفیت زندگی خوبی برخوردارند، امید به بهبود و ادامۀ زندگی نیز در آنان بالاتراز سایرین می‌باشد و هرچه امید به زندگی بالاتر، کیفیت زندگی نیز مطلوب تر خواهد بود در نتیجه این بیماران نسبت به تحمل فشارها و استرس‌های ناشی از درمان بیماری پاسخ مناسبتری از خود نشان می‌دهند و درمان‌های ارائه شده را بهتر پذیرفته و به آن عمل می‌کنند. لذا بهتر است برای این بیماران کلاس‌های آموزشی در جهت ارتقاء کیفیت زندگی  و امید به زندگی آنها انجام شود.

Title: مقایسه اثر مصرف نوشابه ورزشی با محلول قندی بر سطح اسیدلاکتیک دانشجویان بعد از فعالیت‌بی‌هوازی

http://tbj.ssu.ac.ir/article-1-2207-fa.html

تغذیه مناسب پایه و اساس عملکرد جسمانی را تشکیل می دهد. امروزه نوشیدنی های متنوع ورزشی در بازار وجود دارد و ورزشکاران با مقادیر مختلف از آنها در جهت بهبود عملکرد ورزشی استفاده                              می کنند.نوشابه‌های ورزشی همیشه موضوع مورد علا‌قه جوانانی بوده‌اند کهبه ورزش اهمیت خاصی می‌دهند. هدف آن بررسی اثر مصرف نوشابه ورزشی با محلول قندی بر سطح اسید لاکتیک خونی دانشجویان پسر رشته تربیت بدنی دانشگاه یزد بود. روش بررسی: این تحقیق از نوع نیمه تجربی طراحی شده بود و بدین منظور 10 نفر از دانشجویان پسر رشته تربیت بدنی دانشگاه یزد (میانگین سن 7/0+35/19سال، قد 13/3+25/171 سانتی‌متر، میانگین وزن7±1/63 کیلوگرم) به صورت داوطلبانه انتخاب شدند. فعالیت بی‌هوازی آزمودنی ها شامل آزمون RAST می‌باشد که نمونه ها، آزمون را سه دفعه و با فاصله زمانی 48 ساعت از یکدیگر انجام دادند. این گروه 10 نفره به صورت دوسویه کور، 40 دقیقه قبل از اجرای فعالیت بی‌هوازی نوشابه ورزشی ایزوتونیک (داینامین)، محلول قندی ساده و یا دارونما داده شد. قبل و بعد از اجرای فعالیت بی‌هوازی از آزمودنی‌ها نمونۀ خونی گرفته شد. به منظور تجزیه و تحلیل داده‌ها از آزمون  tمستقل استفاده شد. نتایج نشان داد که مصرف نوشابه ورزشی نسبت به دارونما و محلول قندی ساده باعث کاهش معنی‌داری در میزان سطح اسیدلاکتیک آزمودنی‌ها شده است. به طور کلی، به نظر می‌رسد مصرف نوشیدنی ورزشی داینامین می‌تواند سطح اسید لاکتیک خون را در حین فعالیت‌های بی‌هوازی کاهش دهد و احتمالا به بهبود عملکرد منجر شود.

Title: بررسی میزان آلودگی قارچی در آردهای توزیع‌شده در نانوایی‌های شهر یزد

http://tbj.ssu.ac.ir/article-1-2208-fa.html

مقدمه:گندم از مهم‌ترین غلات در ایران می‌باشد و نان تهیه شده از آرد گندم از مهم‌ترین ماده غذایی در جیره غذایی انسان در کشور می‌باشدکه در صورت آلوده بودن اولیه گندم و هم چنین نگهداری و انبار آرد در شرایط نامطلوب محیطی می‌تواند باعث آلودگی آرد به قارچ‌های سمی و به ویژه قارچ‌های مولد آفلاتوکسین شود. هدف از انجام مطالعه حاضر بررسی میزان و نوع آلودگی‌های قارچی در آردهای توزیع شده در نانوایی‌های شهر یزد بوده است. روش بررسی: در این مطالعه توصیفی– مقطعی، نمونه‌های آرد از تعداد 80 نانوایی و هم چنین تعداد 20 نمونه از نمونه‌های آرد تولیدی 4 کارخانه‌ آرد شهر یزد به طور خوشه‌ای و تصادفی تهیه شد. پس از اندازه گیری میزان رطوبت، رقتهای2- 10تا6- 10 نمونه ها برروی محیط کشت سابورودکستروز آگار کشت داده و با براورد تعداد کلنی قارچ‌های جدا شده، جنس و گونه آن ها از روی ساختمان ماکروسکوپی و میکروسکوپی تشخیص داده شدند. اطلاعات را با استفاده از نرم افزار آماری SPSS و به بکارگیری آمارهای توصیفی مانند میانگین، انحراف معیارو هم چنین با آزمون‌های مناسب آماری مانند Chi square test آنالیز آماری انجام گرفت. سطح اطمینان 95% معنی‌دار در نظر گرفته می‌شود. یافته‌ها: مجموعا تعداد2223 کلنی قارچ از 11 جنس و گونه مختلف قارچ از نمونه‌های آرد  مورد مطالعه جدا و شناسایی شدند که تنها 7% نمونه‌های مورد بررسی دارای آلودگی قارچی بیشتر از میزان مجاز بودند. قارچ‌های پنیسیلیوم، کلادوسپوریوم، فوزاریوم، آسپرژیلوس فلاووس و آسپرژیلوس نایجر از شایعترین قارچ‌های و قارچ‌های آلترناریا، ورتیسلیوم، ریزوپوس و فوما با کمترین آلودگی جدا شده از نمونه‌های آرد مورد بررسی در این مطالعه می‌باشند. نتیجه‌گیری: نتایج مطالعه حاضر در مقایسه با مطالعات مشابه بیانگر وضعیت نسبتا مطلوب آردهای توزیع شده در نانوایی‌ها شهر یزد ازنظر میزان آلودگی قارچی در مقایسه با سایر مطالعات مشابه می‌باشد.

Title: بررسی رابطه بین صمیمیت و میل‌جنسی با نشانه‌های افسردگی و رضایت زناشویی در زنان 3ماه تا 2 سال پس از زایمان

http://tbj.ssu.ac.ir/article-1-2209-fa.html

چکیده مقدمه: هدف پژوهش حاضر بررسی رابطه‌ بین میل جنسی و صمیمیت با نشانه‌های افسردگی و رضایت زناشویی در زنان سه ماه تا دو سال پس از تولد نوزاد در شهرستان بندر ماهشهر بود. روش بررسی: پژوهش حاضر، یک مطالعه مقطعی از نوع همبستگی بود، که در اردیبهشت سال 1393بر روی 107 زن مراجعه‌کننده به مراکز بهداشتی شهرستان ماهشهر که دارای فرزند بین سنین سه ماه تا دو سال بودند، انجام شد. اعضای نمونه به‌صورت در دسترس انتخاب و سپس پرسشنامه‌های میل‌جنسی(SIDI)، صمیمیت(PAIR)، افسردگی(CES-D) برای هر کدام تکمیل گردید. داده‌های جمع‌آوری شده با استفاده از آزمون همبستگی پیرسون و رگرسیون چند‌متغیری مورد تجزیه و تحلیل قرار گرفتند. نتایج: بین صمیمیت و میل‌جنسی با نشانه‌های افسردگی رابطه‌ منفی(01/0P≤.) و بین صمیمیت و میل‌جنسی با رضایت زناشویی رابطه ی مثبت (01/0P≤.) به‌دست آمد. متغیر‌های صمیمیت و میل جنسی پیش‌بینی‌کننده‌های معنی‌داری برای نشانه‌های افسردگی و رضایت زناشویی بعد از زایمان بودند و 62 درصد از تغییرات، نشانه‌های افسردگی و 63 درصد رضایت زناشویی را تبیین کردند. نتیجه‌گیری:یافته‌ها نشان دادند که بعد از تولد نوزاد زمانی که زایمان تغییراتی را در زندگی زوج‌‌ها به‌وجود می‌آورد و رضایت زناشویی و خلقیات زنان تحت تأثیر قرار می‌گیرد می‌توان از صمیمیت و میل‌جنسی برای پیش‌بینی افسردگی و رضایت زوج‌ها استفاده کرد.   این مقاله بر گرفته از پایان‌نامه کارشناسی ارشد رشته مشاوره و راهنمایی دانشگاه علوم تحقیقات خوزستان نگاشته شده است.
